# CASE REPORT Anomalies Associated With Congenitally Corrected Transposition of Great Arteries: Expect the Unexpected

**Published:** 2013-01-24

**Authors:** Valay Parikh, Masood A. Shariff, Faisal B. Saiful, Syed Bilal Rizvi, Nikhil Goyal, Kourosh T. Asgarian, Joseph T. McGinn, Thomas Snyder, Vijay A. Singh

**Affiliations:** ^a^Departments of Internal Medicine; ^b^Cardiothoracic Surgery; ^c^Cardiology, and; ^d^Radiology, Staten Island University Hospital, Staten Island, NY

## Abstract

**Objective:** Congenitally corrected transposition of great arteries (CCTGA) is characterized by atrioventricular and ventriculoarterial discordance. Characterizations of these anomalies are important because they may influence surgical approach and management. **Methods:** We present a case of newly diagnosed CCTGA at the age of 50. He presented with sudden onset of shortness of breath for the first time and was diagnosed with CCTGA. Echocardiogram, magnetic resonance imaging, and cardiac catheterization were utilized to elucidate the pathology. **Results:** Intraoperatively, patient's CCTGA and ventricularization of the right ventricle were confirmed. The severe systemic atrioventricular valve regurgitation was replaced with a bioprosthetic valve (Medtronic Mosaic No. 29) with placement of epicardial ventricular leads for possible future placement of automatic implantable cardioverter defibrillators. Pathology report confirmed a degeneration of the systemic atrioventricular valve. **Conclusions:** Significant coronary artery anomalies have also been described in literature with CCTGA. The variances encountered in this case are excellent examples of the intricacies associated in diagnosis and surgical care in patients with CCTGA.

Congenitally corrected transposition of great arteries (CCTGA) is characterized by atrioventricular (AV) and ventriculoarterial (VA) discordance. It has been shown to be associated with different anomalies such as ventricular septal defect (VSD), pulmonary stenosis, valvular abnormalities, and coronary anomalies. Characterizations of these anomalies are important as they may influence surgical approach and management. Here, we present a case where CCTGA that was diagnosed for the first time at the age of 50. We also found anomalous coronary circulation, to our knowledge, which has never been described in the literature. In addition, we point out certain salient features in this case, which may alter the management.

## METHOD/CASE PRESENTATION

A 50-year-old man with no prior medical history was admitted to our hospital for worsening dyspnea on exertion for several weeks. He denied any chest pain, shortness of breath at rest, orthopnea, paroxysmal nocturnal dyspnea, or any palpitations. His family and social histories were noncontributory. His physical examination including vitals was within normal limits, except a grade 3/6 holosystolic murmur over the precordium. Initial workup including electrocardiogram were normal. Echocardiogram, magnetic resonance imaging, and cardiac catheterization were utilized to elucidate the pathophysiology, and surgical intervention was undertaken to replace the valve.

## RESULTS

**Figure 1:**
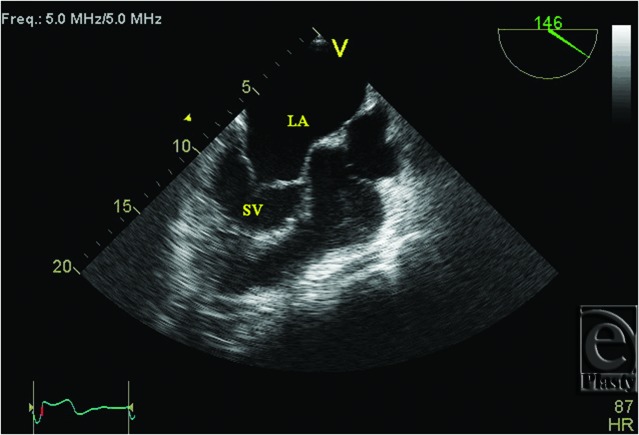
Transesophageal echocardiogram showing hypertrophied SV and dilated LA. LA indicates left atrium; SV, systemic ventricle.

**Figure 2:**
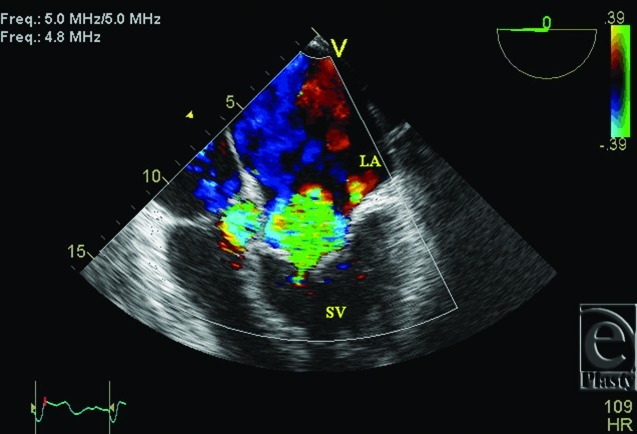
Transesophageal echocardiogram color Doppler showing severe systemic atrioventricular valve regurgitation.

**Figure 3:**
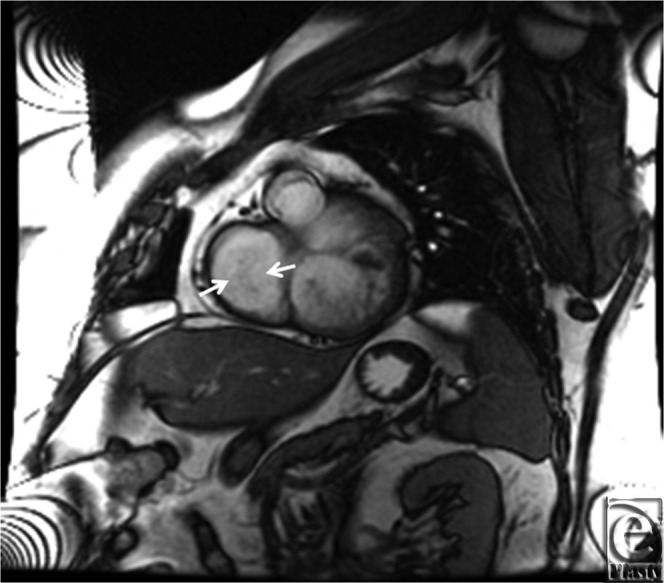
MRI cine SSFP short axis showing a bileaflet valve (white arrows) with in a morphologic left ventricle. This ventricle is noted to be anterior and supports the pulmonary circulation. MRI indicates magnetic resonance imaging.

**Figure 4:**
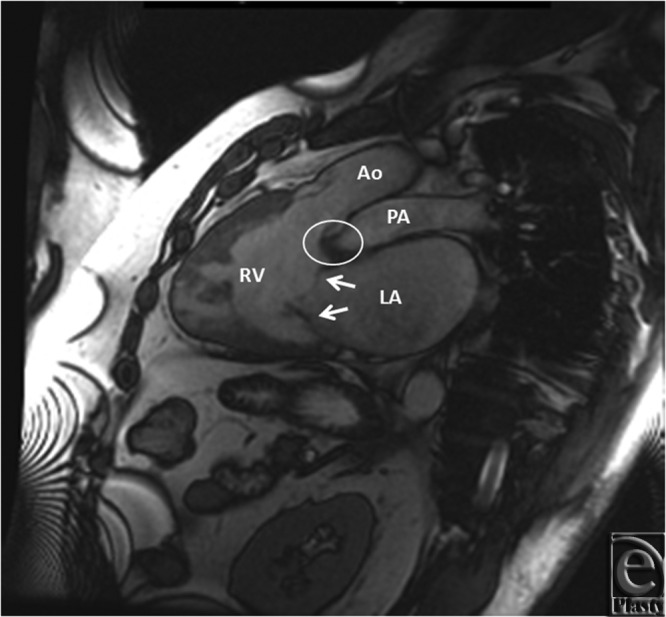
Double-oblique SSFP from the left side showing (1) infundibulum (circle), which conformed the right ventricular morphology. (2) White arrows show 2 of the 3 valve leaflets in the left-sided tricuspid valve. Ao indicates Aorta; LA, left atrium; PA, pulmonary artery; RV, right ventricle. White arrows indicate tricuspid leaflets and white oval indicate infundibulum.

**Figure 5:**
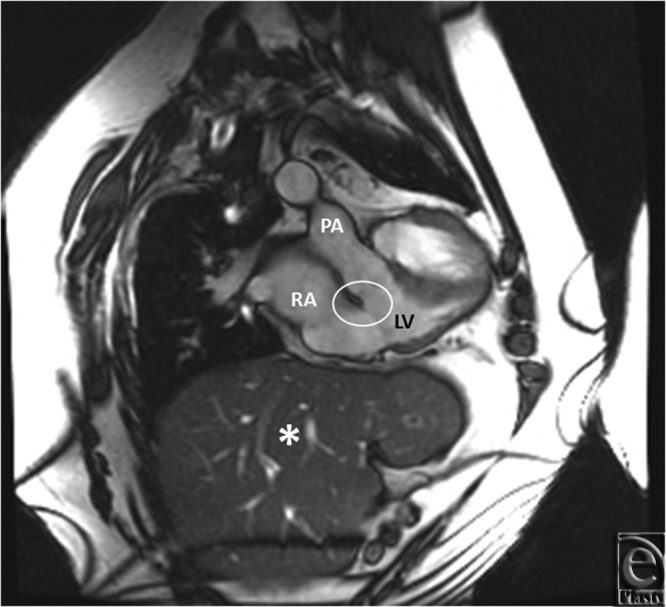
Double-oblique SSFP showing no infundibulum and fibrous continuity of atrioventricular and ventriculoarterial valves consistent with left ventricular morphology. LV indicates left ventricle; PA, pulmonary artery; RA, right atrium. White oval indicates infundibulum asterisk (*) indicates liver.

**Figure 6:**
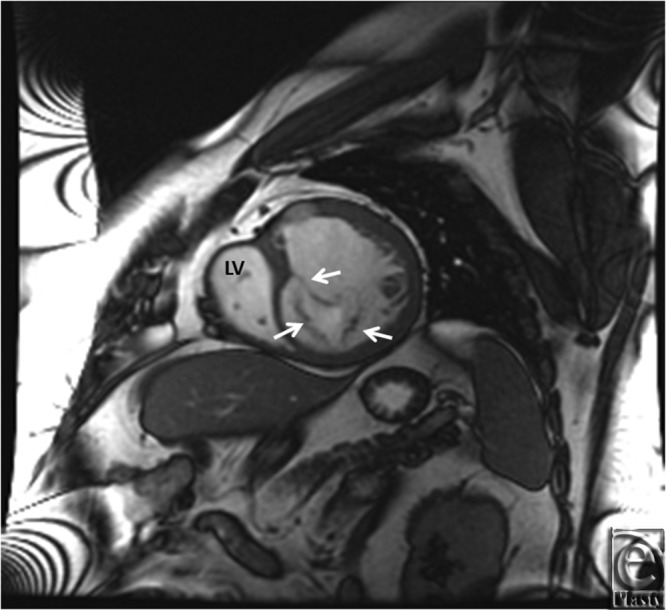
MRI SSFP showing trileaflet valve between left atrium and morphological right ventricle. LV indicates left ventricle; MRI, magnetic resonance imaging. White arrows indicate trileaflet valve.

**Figure 7:**
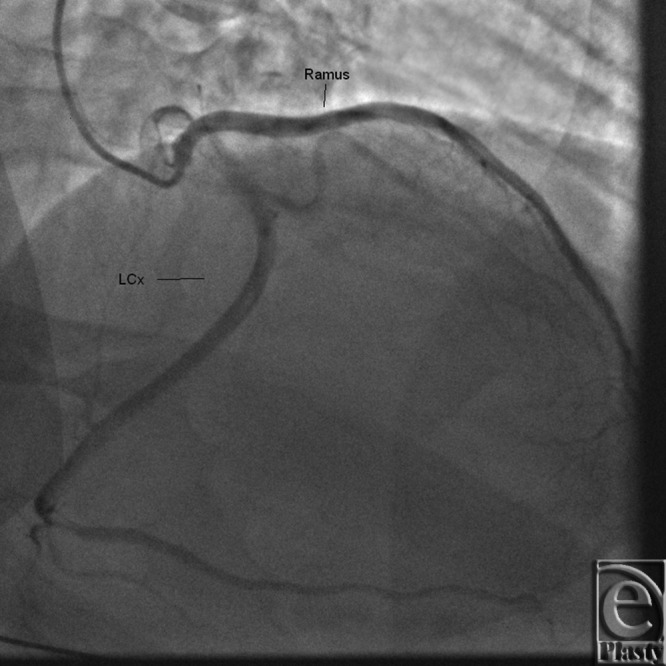
Coronary angiogram showing origins of left circumflex (LCx) and ramus intermedius from different ostia on left coronary cusp.

**Figure 8:**
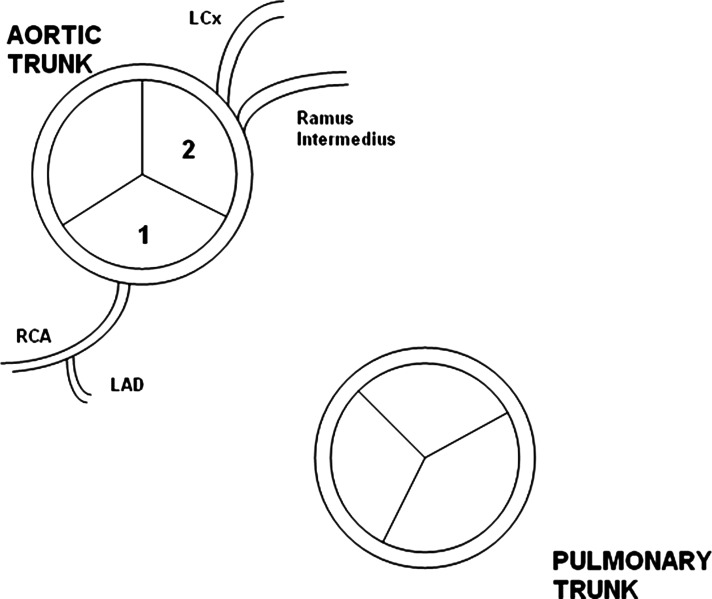
Schematic diagram showing anomalous origin in our case. RCA and LAD originating from an ostium on right coronary cusp, while Ramus Intermedius and LCx originating from separate ostia on left coronary cusp. LAD, left anterior descending; LCx, left circumflex; RCA, right coronary artery.

**Figure 9:**
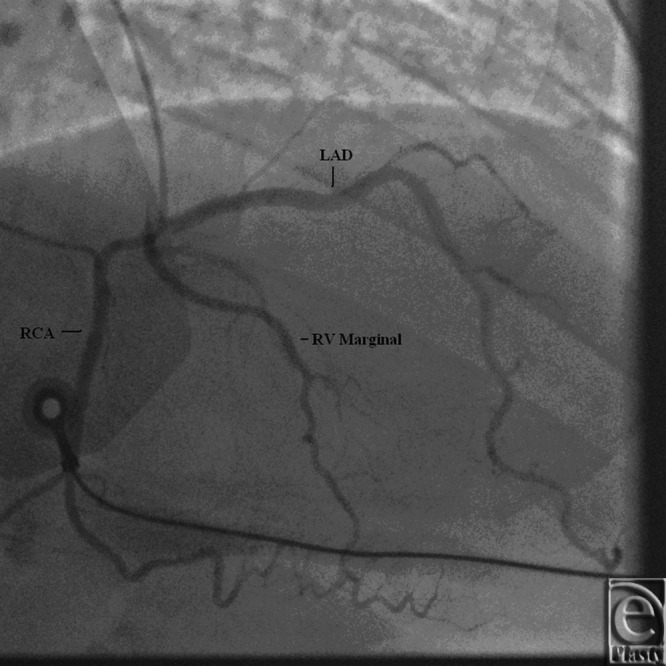
Coronary angiogram showing origin of right coronary artery, RV marginal, and LAD artery from right coronary cusp ostium. LAD indicates left anterior descending; RCA, right coronary artery; RV, right ventricle.

Initial 2D echocardiogram showed severe mitral valve regurgitation with a left ventricular ejection fraction of 35%. Furthermore, the valvular anatomy appeared peculiar; based on right ventricle (RV) and left ventricle morphology, CCTGA was suspected. A transesophageal echocardiography (TEE) was performed for further evaluation. It confirmed our diagnosis of CCTGA. Also, the (TEE) showed a hypertrophied RV and severe systemic AV (SAV) valve regurgitation (Figs 1 and 2). There was no evidence of an atrial septal defect, VSD, or patent ductus arteriosus. An MRI (magnetic resonance imaging) was performed to elucidate the anatomy. It affirmed the diagnosis of CCTGA with a trileaflet SAV that demonstrated severe regurgitation from the systemic ventricle (SV) to left atrium (Figs 3-6). Because of the patient's symptoms and severity of the regurgitation of the SAV, we decided to perform SAV replacement. Preoperative cardiac catheterization showed left dominant coronary circulation with left circumflex and a ramus intermedius originating from left cusp through separate ostia (Figs 7 and 8). Left anterior descending artery originated from proximal right coronary artery through a single ostium from right coronary cusp (Figs 8 and 9). No obstructive coronary heart disease was found and the patient proceeded to surgery.

Intraoperatively, CCTGA and ventricularization of the RV were confirmed. The SAV was replaced with a bioprosthetic valve (Medtronic Mosaic No. 29) with placement of epicardial ventricular leads for possible future placement of automatic implantable cardioverter defibrillator. Pathology report confirmed degeneration of the SAV valve. The patient's postoperative course was unremarkable. He was eventually discharged home in stable condition, 4 days after the conclusion of the procedure.

## DISCUSSION

CCTGA is a rare congenital heart defect (CHD) with estimated prevalence of less than 1% of all CHD.^1^ It is characterized by AV and VA discordance. In addition to that, it has been seen with varied congenital anomalies such as VSD, pulmonary stenosis, valvular malformation, and conduction system dysfunction.^2^^-^4 Eventually, clinical presentation, progression of disease, and the effect of systemic pressure on the functional SV will determine prognosis. Apart from valvular replacement, interventions like an atrial switch with a Rastelli procedure are usually performed to correct these anomalies and relieve symptoms.

Significant coronary artery anomalies have also been described in literature with CCTGA.^5^^-^7 In a study performed by Ismat et al^8^ with autopsy on pediatric hearts having CCTGA, coronary circulation was shown to be more consistent as compared to other CHD.^8^^-^12 Such anomalies should be kept in mind, when performing corrective surgery.^13^ Coronary circulation can be delineated with the help of coronary angiography or MRI. While coronary angiography has an added benefit in evaluation of coronary circulation and intraluminal abnormalities, an MRI is beneficial for further characterization of anatomical structures and to identify other associated anomalies. In our case, the aberrations were delineated with coronary angiography. The patient had a common origin for the right coronary artery and left anterior descending artery. Meanwhile, the left coronary cusp gave off 2 branches, namely, left circumflex artery and a ramus intermedius through separate ostia. This anomaly has never been described before in literature. Although the coronary anatomy is not directly involved in surgical correction, variants must be kept in mind during manipulation especially when undergoing corrective surgery.

Apart from that, our patient had certain other rare interesting findings. No VSD, atrial septal defect, or patent ductus arteriosus was confronted, which is reported in just 15% of patients.^14^ These anomalies can help with the function of the heart, especially when performing a corrective procedure. Specifically, they can aid in unloading new intracardiac volume as a result of valvular competency. In our particular case, these anomalies were not evident, which lead us to question if corrective surgery would be tolerated. For example, correction of SAV could have lead to volume overload for the SV, in this case the morphologic RV. With no associated anomalies, a postsurgical competent SAV could place unaccounted strain on the SV. In general, there is always a question, if the RV can adapt to systemic pressure. If the answer to that question is no, then SV failure will ensue. Fortunately, in our case, the SV was able to tolerate the hemodynamic burden without any undue complications.

Although in the literature, older and unoperated patients are reported, with a median age of 40 years at death in patients both who have undergone operation and who have not, our patient was a 50-year-old man and represents an important exception.^15^^,^^16^ We feel the variances we encountered in this case are excellent examples of the intricacies associated with CCTGA. Because of wide clinical spectrum, it is prudent to anticipate unexpected abnormalities, which may affect the approach and outcomes.
